# Additive Mixture Effects of Estrogenic Chemicals in Human Cell-Based Assays Can Be Influenced by Inclusion of Chemicals with Differing Effect Profiles

**DOI:** 10.1371/journal.pone.0043606

**Published:** 2012-08-17

**Authors:** Richard Mark Evans, Martin Scholze, Andreas Kortenkamp

**Affiliations:** Institute for the Environment, Brunel University, Uxbridge, Middlesex, United Kingdom; Indian Institute of Toxicology Reserach, India

## Abstract

A growing body of experimental evidence indicates that the *in vitro* effects of mixtures of estrogenic chemicals can be well predicted from the estrogenicity of their components by the concentration addition (CA) concept. However, some studies have observed small deviations from CA. Factors affecting the presence or observation of deviations could include: the type of chemical tested; number of mixture components; mixture design; and assay choice. We designed mixture experiments that address these factors, using mixtures with high numbers of components, chemicals from diverse chemical groups, assays with different *in vitro* endpoints and different mixture designs and ratios. Firstly, the effects of mixtures composed of up to 17 estrogenic chemicals were examined using estrogenicity assays with reporter-gene (ERLUX) and cell proliferation (ESCREEN) endpoints. Two mixture designs were used: 1) a ‘balanced’ design with components present in proportion to a common effect concentration (e.g. an EC_10_) and 2) a ‘non-balanced’ design with components in proportion to potential human tissue concentrations. Secondly, the individual and simultaneous ability of 16 potential modulator chemicals (each with minimal estrogenicity) to influence the assay outcome produced by a reference mixture of estrogenic chemicals was examined. Test chemicals included plasticizers, phthalates, metals, PCBs, phytoestrogens, PAHs, heterocyclic amines, antioxidants, UV filters, musks, PBDEs and parabens. In all the scenarios tested, the CA concept provided a good prediction of mixture effects. Modulation studies revealed that chemicals possessing minimal estrogenicity themselves could reduce (negatively modulate) the effect of a mixture of estrogenic chemicals. Whether the type of modulation we observed occurs in practice most likely depends on the chemical concentrations involved, and better information is required on likely human tissue concentrations of estrogens and of potential modulators. Successful prediction of the effects of diverse chemical combinations might be more likely if chemical profiling included consideration of effect modulation.

## Introduction

Humans are typically exposed to multiple chemicals with diverse effects [1]. Despite this, experimental studies usually examine binary or ternary combinations. We recently reviewed 173 experimental mixture studies and found that the majority had tested binary combinations and that fewer than one in four studies had examined mixtures with seven or more components [2].

Of the available mathematical concepts for the prediction of mixture effects, concentration addition (CA) has proven the most useful and has been shown to have good predictive power, see reviews by [1], [3]. Ermler *et al.* recently showed that mixtures of 17 anti-androgenic chemicals with varied structural features produced effects *in vitro* that were predictable by CA [4]. However, a study of a similar number of estrogenic chemicals observed small deviations from the predictions made using CA [5]. Silva *et al.* studied five mixtures with from 3 to 16 components in the ESCREEN assay. The effects of two mixtures were accurately predicted by CA, whilst three showed slight overestimation by CA. It was hypothesised that the deviation was due to increased metabolism of steroidal estrogens and it was suggested that CYP1B1 activation and reduction in steroidal estrogen concentrations could “contribute to the shortfall from [CA]” [5]. This hypothesis was tested by predicting the mixture effect if the steroidal estrogens had been removed by metabolism (i.e. they make no contribution to the overall effect) and comparing this scenario with the observed result. Few other studies have examined mixtures with a similar number of components, and factors that remain to be addressed include the assay used, the number and type of chemical studied and the mixture design. Further studies in this area are required in order to resolve whether predictability by CA should be the default expectation for multicomponent mixtures of estrogens. If so, CA could be routinely applied for the assessment of such mixtures.

In this paper we aimed to address the impact of choice of assay system, the number and nature of included chemicals, the mixture design, and the possibility of effect modulation. Our approach to each of these issues is now described in turn.

### Assay System

To evaluate the possible contribution of the model system, we have compared the predictability of mixture effects in two *in vitro* assays with differing endpoints, the ERLUX and ESCREEN assays. These assays utilize a luminescent reporter gene and a cell proliferation endpoint respectively. The ESCREEN assay has been widely used in the study of environmental estrogens [6], [7] and the ERLUX is one of a number of available reporter gene assays that are becoming increasingly used in the field [8]. Both assays are established *in vitro* assays based on human cell lines, however we had an expectation that the more apical endpoint used in the ESCREEN would provide greater potential for deviations of mixture effects from those predicted compared to the more constrained ERLUX endpoint, which is a luminescent signal indicating activation of an engineered reporter-gene.

### Number of Components

We have chosen to study mixtures with a high number of components (up to 30 components for a mixture of estrogens with potential modulators). We aimed for a high number of components because systematic testing of all possible mixture combinations, for example recursively studying binary, ternary, quaternary etc combinations, is not practical and we considered that the most interesting combinations, from a toxicological scenario, are those of higher numbers of components.

### Type of Chemical

We wished to avoid testing mixtures of only congeneric chemicals, since this does not reflect likely exposure scenarios and is likely to limit the possible effect ‘repertoires’ that can be observed. Chemicals were selected from chemical groups to which human exposure is likely, especially through food, and included: plasticizers including phthalates, metals, polychlorinated biphenyls (PCBs), phytoestrogens, polyaromatic hydrocarbons (PAHs), heterocyclic amines, antioxidants, UV filters, musks, polybrominated diphenylethers (PBDEs) and parabens.

### Mixture Design

We have also studied mixtures designed from two different perspectives. Firstly, we have used a design in which all components were combined in proportion to their potency. This was achieved by choosing a mixture ratio in proportion to selected effect concentrations, for example an EC_10_ or EC_25_; we refer to this approach as a “balanced” design since each component is expected to contribute equally to the overall effect of the mixture. The advantage of this design is that the contribution of any one component is equally likely to be evident (as a contribution towards an additive mixture effect or as a deviation from additivity) as any other component, and so the number of possible interactions that could be evident is maximized. The balanced design also eliminates the possibility of performing a mixture experiment in which the combined effect is due almost entirely to the actions of a few or only one component. Secondly, we have used mixtures based on the ratio of the possible human tissue concentration. Accurate, consistent data on human tissue levels is not generally available, but the literature contains some information that can be employed with caution. We refer to this approach as a “non-balanced” design, since the design is not based on the potency of each component, and the mixture effect may be dominated by one or several of the mixture components, if that is the observed human exposure situation. The purpose of this design is to allow the testing of mixtures with a composition that is arguably more realistic that the equieffective design, since it is unlikely that humans are exposed to chemicals in a fixed proportion to their potency on any one given endpoint. It is possible to criticise this design because it is based on often incomplete human exposure data, with extrapolation between reported and compared tissue levels, reports derived from different publications, analytical systems and geographical populations. However, even if the underlying ratio is subsequently found to be inappropriate, the test design still represents a ***different*** mixture ratio to that of the balanced design and so in any case constitutes a further test of the CA concept. Because of these caveats, we do not intend to draw strong conclusions about the actual human situation from these experimental observations, but rather propose that they contribute to future experimental designs and serve to test the wider suitability of CA for use in modeling mixture effects.

### Effect Modulation

We recognised the need to study chemicals that were more varied than congeners (see above) and also extended this further to examine the effects of including chemicals that had weak or no estrogenicity themselves, which we termed possible effect modulators. Possible modulators included those that had been reported as estrogenic in the literature but that did not exhibit such behaviour in our experimental systems. An example of this is the carcinogen PhIP, which has been reported to be an estrogen [9] but did not behave as such in our hands [10].

A practical issue in studying effect modulators is that to compare around 15 modulators with around 15 actives, and mixtures thereof, is not practically possible. For example, to test 15 modulators against 15 actives in triplicate, would require 675 experimental runs, and would not even provide any data on possible modulation by combinations of chemicals. Therefore, we employed an approach of screening the individual modulators with low power, and simultaneously testing an equimolar mixture of all modulators. The use of an equimolar design is suitable when the actual effect is not known, e.g. we do not know how, or indeed if, modulation will occur. The data from low power screening and from mixture testing is then evaluated collectively and the mixture result is assessed for its ability to cross-validate the screening results. With this approach we propose that meaningful observations were made using a much more feasible 20 plates and that the approach could be used or extended for similar situations.

## Materials and Methods

### Ethics Statement

The two human cell lines used in this work were obtained commercially or as a gift, and their origins were previously described in publications from other groups: the ESCREEN assay uses cloned MCF-7 cells (described in [6]) that were gifted to us; the ERLUX assay uses T47D-KBluc cells (described in [11]) that were purchased commercially.

### Chemicals

Cell culture reagents were purchased from Invitrogen (Paisley, UK). Test compounds were obtained as listed in Table 1.

**Table 1:** Details of test chemicals.

Chemical name	Abbreviation	Supplier (Catalogue number)	CAS number
2,2′,3,4,4′,5,5′-heptachloro biphenyl (PCB #180)	PCB180	UltraScientific (RPC-094)	35065-29-3
2,2′,4,4′,5,5′-hexachloro biphenyl (PCB #153)	PCB153	UltraScientific (RPC-047)	35065-27-1
2,4′-dichlorobiphenyl (PCB #8)	PCB008	UltraScientific (RPC-089)	34883-43-7
2-amino-1-methyl-6-phenylimidazo [4,5-b] pyridine	PhIP	Apollo (OR1700T), MP Biomedicals (154190),Toronto Research Chemicals (A617000)	105650-23-5
2-Amino-3,8-dimethyl imidazo [4,5-f] quinoxaline	MeIQx	Apollo (ORO660T)	77500-04-0
3,3′,4,4′,5-pentachlorobiphenyl (PCB #126)	PCB126	LGC (ERM AC821)	57465-28-8
4-methylbenzylidene camphor	4MBC	Merck (1.05383.0100)	36861-47-9
Benzo [a] pyrene	BaP	Sigma SUPELCO (48564)	50-32-8
Benzophenone-3	BP3	Sigma Aldrich (H36206)	131-57-7
Bisphenol A	BPA	Sigma (239658)	80-05-7
Brominated diphenyl ether-100	BDE100	LGC (CIL-EO-4194)	189084-64-8
Butyl benzyl phthalate	BBP	Sigma (36927)	85-68-7
Butylated hydroxyl anisole	BHA	Sigma (W218308)	25013-16-5
Butylated hydroxytoluene	BHT	Sigma (W218405)	128-37-0
Butylparaben	BUTYLP	Sigma (H9503)	94-26-8
Cadmium chloride	CdCl2	Sigma (C3141)	7790-78-5
Coumestrol	COU	Sigma Fluka (27883)	479-13-0
Di butyl phthalate	DBP	Sigma (36736)	84-74-2
Di ethyl hexyl phthalate	DEHP	Sigma (36735)	117-81-7
Di ethyl phthalate	DEP	Sigma (36737)	84-66-2
Enterolactone	ENL	Sigma Fluka (45199)	78473-71-9
Estradiol	E2	Sigma (E2758)	50-28-2
Ethinyl estradiol	EE2	Sigma (E4876)	57-63-6
Fluoranthene	FLUOR	Sigma Riedel-de-Haen (45504)	206-44-0
Galaxolide (HHCB)	GAL	LGC (DEOMUS-01)	1222-05-5
Genistein	GEN	Lancaster (L14171)	446-72-0
Lead nitrate	Pb (NO3)2)	Sigma (203580)	10099-74-8
Mercury chloride	HGCl2	Sigma (429724)	7487-94-7
Methylparaben	METHYLP	Acros Organics (126961000)	99-76-3
Naringenin	NAR	Sigma Fluka (71155)	67604-48-2
Propylparaben	PROPYLP	Sigma (P53357)	94-13-3
Tonalide (AHTN)	TON	LGC (DE-MUS-02)	21145-77-7

### Estrogenicity Assay, Reporter Gene Endpoint (ERLUX)

T47D-KBluc cells were obtained from the ATCC and the protocol established by the depositing authors was followed [8]. Cells were routinely grown in RPMI media (with 10% foetal calf serum (FCS)). For seven days prior to experiments, cells were maintained in low estrogen conditions by the use of pre-assay media (RPMI, 10% charcoal-dextran stripped FCS, no antibiotics). For experiments, cells were seeded in white polystyrene 96 well plates at a density of 10,000 cells/well and allowed to attach for 24 hours before removal of media, and application of test chemicals. Test chemicals were dissolved in ethanol to give stock solutions of millimolar concentrations. Test and control solutions were obtained by dilution of ethanolic stocks in dosing media (phenol red-free RPMI, 5% charcoal-dextran stripped FCS, no antibiotics), and in all cases the final concentration of ethanol was 0.5%. The positive control was 1 nM estradiol. Positive and vehicle controls were run as eight replicate wells per plate, and compounds were tested in a dilution series comprising eight concentrations, each concentration tested in triplicate. As recommended by Wilson *et al.* two additional controls were also included on each plate: 1) vehicle control plus an antiestrogen, ICI 182,780 (1 µM), and 2) positive control plus ICI 182,780 (1 µM). These additional controls were used to monitor the background level of estrogenicity, and full experiments were excluded if vehicle showed high levels of estrogenicity or if the positive could not be suppressed by the antiestrogen (data not shown). A crude measure of toxicity was provided by comparing values for treatments that were not positive, with the value of the vehicle control. Toxicity would be expected to decrease these small (but non-zero) values towards zero, and this was not observed for any of the tested chemicals (data not shown). 24 hours after application of test and control solutions, a volume of Steady-Glo assay reagent (Promega) equal to the volume of culture media was added and plates were incubated for ten minutes, with shaking, to allow for cell lysis. Plates were then loaded into a plate reader (FLUOstar Optima, BMG Labtech) and incubated for a further ten minutes in the dark, followed by measurement of luminescence. To reduce variation, the temperature of the plate reader chamber was maintained at 27°C throughout.

### Estrogenicity Assay, Mitogenic Endpoint (ESCREEN)

The ESCREEN assay was performed using cloned MCF-7 cells (described in [6], gifted from A. Soto, Boston) and the established ESCREEN method [6] was followed using an adapted 96-well format [11]. Cells were cultured in DMEM (5% FCS). For experiments, cells were seeded in clear polystyrene 96 well plates at a density of 2,500 cells/well and allowed to attach for 24 hours before washing with rinse media (phenol red-free DMEM, no supplements). Estrogen deprivation (by use of charcoal-dextran stripped serum and removal of phenol red) was not used prior to seeding because this results in an almost complete lack of attachment of cells. Test chemicals were dissolved in ethanol to give stock solutions of millimolar concentrations. Test and control solutions were diluted prior to application in dosing media (phenol red free DMEM, 10% charcoal-dextran stripped FCS). The final concentration of ethanol was 0.5% in test and control wells. The positive control was 25 nM estradiol and the final concentration of ethanol in all wells was 0.5%. The raw value of vehicle controls was monitored for any indication of increasing background estrogenicity, raw values were typically 0.06–0.08 optical density units (ODU) and experiments were rejected if the vehicle value (averaged per plate) exceeded 0.1 ODU. At all stages, media removal from cells was carried out gently and in a controlled fashion by use of an electronic multichannel pipette set to the lowest speed possible. Controls were run as eight replicate wells per plate and compounds were tested in a dilution series comprising eight concentrations, each concentration tested in two replicate wells per plate. The plate layout was designed to reduce variation due to evaporation and spreading of test chemicals, and had been previously optimised in the laboratory [11]. After application of test solutions, plates were incubated for 120 hours before fixation with 10% trichloroacetic acid and sulforhodamine B (SRB) staining to measure protein and allow the indirect quantification of cell number.

**Table 2:** Estrogenicity of individual compounds (ERLUX).

		Concentration Response Function					EC_10_		EC_25_	
Substance (in orderof EC_10_)	RM	θ^∧^_1_	θ^∧^_2_	θ^∧^_3_	θ_min_	θ^∧^_max_	M [CI]		M [CI]	
EE2	G.logit I	125.57	11.01	0.27	0	1.05	6.51E-13	[4.19E-13 – 8.77E-13]	1.32E-12	[9.68E-13 – 1.55E-12]
Estradiol	logit	34.98	3.07	–	0	1.23	6.53E-13	[4.29E-13 – 9.97E-13]	1.45E-12	[1.03E-12 – 2.02E-12]
Coumestrol	logit	26.52	3.26	–	0	1.01	1.58E-9	[1.19E-9 – 2.26E-9]	3.42E-9	[2.50E-9 – 4.90E-9]
Genistein	logit	38.76	5.23	–	0	1.48	1.24E-8	[1.15E-8 – 1.34E-8]	1.96E-8	[1.86E-8 – 2.06E-8]
Bisphenol A	logit	29.24	4.68	–	0	1.44	1.55E-7	[1.39E-7 – 1.73E-7]	2.58E-7	[2.43E-7 – 2.75E-7]
Naringenin	logit	30.45	5.04	–	0	1.14	3.10E-7	[2.57E-7 – 4.74E-7]	5.06E-7	[4.40E-7 – 6.15E-7]
Butylparaben	logit	20.97	3.88	–	0	3.31	4.97E-7	[2.72E-7 – 6.75E-7]	8.81E-7	[6.19E-7 – 1.05E-6]
Benzo [a] pyrene	logit	15.94	2.97	–	0	0.90	8.62E-7	[6.64E-7 – 1.09E-6]	2.06E-6	[1.60E-6 – 2.59E-6]
Propylparaben	logit	21.65	4.22	–	0	2.60	1.30E-6	[1.01E-6 – 1.60E-6]	2.21E-6	[1.86E-6 – 2.54E-6]
4MBC	G.logit I	81.54	17.24	0.12	0	0.82	1.81E-6	[1.34E-6 – 2.86E-6]	5.00E-6	[4.08E-6 – 6.15E-6]
Benzophenone-3 (BP3)	logit	15.69	3.28	–	0	1.77	2.27E-6	[1.46E-6 – 2.88E-6]	4.61E-6	[3.40E-6 – 5.67E-6]
Tonalide	logit	7.74	1.53	–	0	0.28	3.61E-6	[1.74E-6 – 9.63E-6]	2.06E-4	[2.96E-5 – 5.25E-4]
Enterolactone	logit	15.32	3.24	–	0	1.04	3.77E-6	[3.47E-6 – 4.10E-6]	8.18E-6	[7.49E-6 – 9.06E-6]
Galaxolide	logit	18.54	3.82	–	0	0.91	3.93E-6	[3.05E-6 – 5.02E-6]	7.73E-6	[6.68E-6 – 8.77E-6]
BD100	logit	11.34	2.68	–	0	1.72	5.36E-6	[4.46E-6 – 6.65E-6]	1.28E-5	[9.71E-6 – 1.55E-5]
Methylparaben	logit	12.76	3.09	–	0	2.06	8.14E-6	[5.49E-6 – 1.13E-5]	1.71E-5	[1.25E-5 – 2.29E-5]
Fluoranthene	logit	16.72	3.85	–	0	0.16	6.00E-5	[3.70E-5 – 1.22E-4]	–	
*Mixtures with ratio as defined in * *Table 4* *.*										
Mixture 1	logit	13.91	3.24	–	0	1.24	8.94E-6	[7.37E-6 - 1.15E-5]	1.90E-5	[1.75E-5 - 2.25E-5]
Mixture 2	probit	12.18	2.21	–	0	1.97	5.69E-7	[4.91E-7 - 6.67E-7]	9.54E-7	[8.35E-7 - 1.12E-6]

EC_10_, EC_25_: concentration producing 10% and 25% effect, respectively. Values in brackets denote the upper and lower limits of the approximate 95% confidence interval; the column “RM” indicates the mathematical regression function as defined by [13]; θ^∧^_1_, θ^∧^_2_, θ^∧^_3_, θ^∧^_max_ estimated model parameters, given for concentrations expressed in M (rounded values); θ_min_ were not estimated, but set to 0 relating to the mean value of the negative vehicle controls.

**Table 3:** Estrogenicity of individual compounds (ESCREEN).

		Concentration Response Function					EC_10_		EC_25_	
Substance (by orderof EC_10_)	RM	θ^∧^_1_	θ^∧^_2_	θ^∧^_3_	θ_min_	θ^∧^_max_	M [CI]		M [CI]	
EE2	logit	26.38	2.36	–	0	1.14	6.35E-13	[5.63E-14 – 1.53E-12]	1.81E-12	[3.34E-13 – 3.39E-12]
Estradiol	G.logit I	24.30	2.23	1.08	0	1.33	1.12E-12	[6.18E-13 – 1.40E-12]	3.12E-12	[2.46E-12 – 3.80E-12]
Coumestrol	G.logit I	19.59	0.87	394769	0	1.31	1.56E-9	[9.45E-10 – 2.18E-9]	5.00E-9	[3.79E-9 – 6.37E-9]
Genistein	logit	17.63	2.37	–	0	0.93	4.61E-9	[1.68E-9 – 1.34E-8]	1.36E-8	[6.45E-9 – 2.95E-8]
Bisphenol A	G.logit I	19.06	2.69	2.251	0	1.21	4.52E-8	[3.41E-8 – 5.81E-8]	8.18E-8	[6.59E-8 – 1.04E-7]
Naringenin	logit	16.78	2.96	–	0	1.10	3.60E-7	[2.53E-7 – 4.97E-7]	8.33E-7	[6.82E-7 – 1.09E-6]
Butylparaben	logit	16.61	3.01	–	0	1.01	5.71E-7	[3.60E-7 – 8.35E-7]	1.32E-6	[9.84E-7 – 1.77E-6]
BDE100	G.logit I	90.69	18.09	0.095	0	0.71	7.04E-7	[5.37E-7 – 9.43E-7]	2.41E-6	[2.01E-6 – 2.77E-6]
4MBC	logit	17.65	3.20	–	0	0.63	8.99E-7	[6.52E-7 – 1.12E-6]	2.21E-6	[1.49E-6 – 3.05E-6]
Propylparaben	logit	17.05	3.22	–	0	1.09	9.97E-7	[8.84E-7 – 1.10E-6]	2.16E-6	[1.96E-6 – 2.34E-6]
Benzophenone-3	G.logit I	38.08	7.86	0.23	0	0.63	1.30E-6	[6.00E-7 – 2.30E-6]	4.31E-6	[3.05E-6 – 5.29E-6]
Tonalide	logit	15.54	2.95	–	0	0.46	1.97E-6	[1.30E-6 – 2.83E-6]	6.13E-6	[4.70E-6 – 8.21E-6]
Enterodiol	Weibull	20.70	4.08	–	0	0.45	3.85E-6	[1.32E-6 – 4.38E-6]	7.45E-6	[6.10E-6 – 8.02E-6]
Enterolactone	Weibull	19.22	4.05	–	0	1.39	4.15E-6	[2.02E-6 – 5.00E-6]	7.22E-6	[5.50E-6 – 8.27E-6]
Galaxolide	logit	16.10	3.39	–	0	0.69	5.39E-6	[3.98E-6 – 7.92E-6]	1.23E-5	[8.96E-6 – 1.82E-5]
Methylparaben	Weibull	12.19	2.77	–	0	0.79	7.64E-6	[5.56E-6 – 9.97E-6]	1.80E-5	[1.42E-5 – 2.12E-5]
Fluoranthene	logit	17.10	4.05	–	0	0.38	3.39E-5	[3.34E-5 – 4.19E-5]	8.85E-5	[8.45E-5 – 9.85E-5]
*Mixtures with ratio as defined in * *Table 4* *.*										
Mixture 3a	logit	16.39	3.45	–	0	0.99	4.07E-6	[3.40E-6 - 4.48E-6]	8.49E-6	[7.98E-6 - 9.03E-6]
Mixture 3b	logit	20.15	4.22	–	0	1.03	5.00E-6	[3.67E-6 - 6.12E-6]	9.07E-6	[7.54E-6 - 1.08E-5]
Mixture 3c	logit	18.16	3.83	–	0	0.93	5.00E-6	[3.67E-6 - 6.13E-6]	9.79E-6	[8.64E-6 - 1.09E-5]
Mixture 3d	logit	15.44	3.15	–	0	0.80	3.07E-6	[2.63E-6 - 3.41E-6]	7.15E-6	[6.01E-6 - 9.04E-6]
Mixture 4	logit	11.44	2.11	–	0	1.51	2.12E-7	[1.56E-7 - 2.81E-7]	6.52E-7	[5.33E-7 - 8.02E-7]

EC_10_, EC_25_: concentration provoking 10% and 25% effect, respectively. Values in brackets denote the upper and lower limits of the approximate 95% confidence interval; the column “RM” indicates the mathematical regression function as defined by [13]; θ^∧^_1_, θ^∧^_2_, θ^∧^_3_, θ^∧^_max_ estimated model parameters, given for concentrations expressed in M (rounded values); θ_min_ were not estimated, but set to 0 relating to the mean value of the negative vehicle controls.

**Table 4:** Test mixtures (ERLUX & ESCREEN).

	Relative proportions (percentages)						
	ERLUX		ESCREEN				
Components	Mixture 1 (17 agents)	Mixture 2 (14 agents)	Mixture 3a (16 agents)	Mixture 3b (15 agents)	Mixture 3c (14 agents)	Mixture 3d (14 agents)	Mixture 4 (13 agents)
EE2	4.09E-07%	–	0.00000031%	–	–	0.000002%	–
Estradiol	4.19E-07%	6.90E-05%	0.00000163%	0.00000163%	–	0.000003%	0.000069%
Coumestrol	0.00105%	0.138035%	0.00312123%	0.00312123%	0.00312123%	0.005348%	0.13812%
Genistein	0.00818%	0.345087%	0.01492644%	0.01492644%	0.01492644%	0.026514%	0.34530%
Bisphenol A	0.12071%	1.380347%	0.05328694%	0.05328694%	0.05328694%	0.092497%	1.38121%
Naringenin	0.28511%	13.803470%	0.54038307%	0.54038307%	0.54038308%	0.869146%	13.812%
Butylparaben	0.33653%	0.690174%	0.96699631%	0.96699631%	0.96699633%	1.538250%	0.69060%
4MBC	1.34203%	5.521388%	1.96523825%	1.96523825%	1.96523828%	2.391306%	5.52482%
Propylparaben	1.31881%	0.690174%	1.43773784%	1.43773784%	1.43773787%	2.287083%	0.69060%
Benzophenone-3	1.13717%	69.017352%	3.42226015%	3.42226016%	3.42226021%	5.443963%	69.060%
Tonalide	3.27036%	–	4.13837283%	4.13837285%	4.13837291%	–	–
Enterodiol	–	–	4.99888135%	4.99888136%	4.99888144%	–	–
Enterolactone	3.73626%	1.380347%	4.81072053%	4.81072054%	4.81072062%	4.503069%	1.38121%
Galaxolide	2.95743%	–	5.80157693%	5.80157695%	5.80157704%	–	–
Methylparaben	6.83250%	6.901735%	13.36730927%	13.36730931%	13.36730953%	21.264058%	6.90603%
Fluoranthene	74.1155%	0.069017%	58.47918693%	58.47918711%	58.47918807%	58.976087%	0.06906%
Benzo [a] pyrene	0.63597%	0.062100%	–	–	–	–	–
BDE100	3.90238%	6.90E-04%	–	–	–	2.602675%	0.00069%

Rounded values given for relative proportions.

**Table 5:** Statistical uncertainty of predicted and observed effect concentrations for mixtures (ERLUX).

Effect level *x*	Effect concentration ECx_mix_ [M]					
	Observed		Predicted by CA		Predicted by IA	
	Mean	95% CI	Mean	95% CI	Mean	95% CI
*Mixture 1∶17 components (ratio as defined in * *Table 4* *)*						
10%	8.94E-6	[7.37E-6 – 1.15E-5]	7.47E-6	[6.61E-6 – 8.55E-6]	1.35E-5	[8.98E-6 – 1.68E-5]
25%	1.90E-5	[1.75E-5 – 2.25E-5]	1.62E-5 – 1.79E-5*		3.17E-5	[2.54E-5 – 3.57E-5]
50%	3.82E-5	[3.22E-5 – 4.64E-5]	2.71E-5 – 3.24E-5*		6.37E-5	[5.50E-5 – 6.94E-5]
*Mixture 2∶14 components (ratio as defined in * *Table 4* *)*						
10%	5.69E-7	[4.91E-7 – 6.67E-7]	3.22E-7	[2.69E-7 – 3.91E-7]	5.41E-7	[4.05E-7 – 7.20E-7]
25%	9.54E-7	[8.35E-7 – 1.12E-6]	6.44E-7 – 6.44E-7*		1.10E-6	[8.91E-7 – 1.43E-6]
50%	1.57E-6	[1.35E-6 – 1.91E-6]	1.98–7 – 1.23E-6*		2.06E-6	[1.72E-6 – 2.53E-6]

CA, Concentration Addition; IA, Independent Action; CI, Confidence Interval. All predictions statistically significant to the observed ECs are shown in bold. *Effect mixture concentration for effect levels higher than the lowest estimated compound maximal model asymptote are extrapolated either (i) by assuming no contribution of this compound to the overall mixture effect (toxic unit equals zero), or (ii) by setting the compounds’ toxic unit to a fixed level equalling the value at the mixture concentration producing an effect of 0.7*θ_max_ (see Table 2). The right side of the interval corresponds to (i) and the left side to (ii), defining the range of possible CA predictions.

**Table 6:** Statistical uncertainty of predicted and observed effect concentrations for mixtures (ESCREEN).

Effect level *x*	Effect concentration ECx_mix_ [M]					
	Observed		Predicted by CA		Predicted by IA	
	Mean	95% CI	Mean	95% CI	Mean	95% CI
*Mixture 3a: 16 components (ratio as defined in * *Table 4* *)*						
10%	4.07E-6	[3.40E-6 – 4.48E-6]	3.71E-6	[2.76E-6 – 4.24E-6]	6.00E-6	[2.80E-6 – 8.21E-6]
25%	8.49E-6	[7.98E-6 – 9.03E-6]	9.36E-6	[8.09E-6 – 1.02E-5]	1.41E-5	[8.98E-6 – 1.82E-5]
50%	1.77E-5	[1.57E-5 – 2.11E-5]	1.75E-05 – 3.24E-5*		2.94E-5	[2.26E-5 – 3.51E-5]
*Mixture 3b: 15 components (ratio as defined in * *Table 4* *)*						
10%	5.00E-6	[3.67E-6 – 6.12E-6]	3.78E-6	[2.98E-6 – 4.32E-6]	6.18E-6	[3.25E-6 – 8.79E-6]
25%	9.07E-6	[7.54E-6 – 1.08E-5]	9.51E-6	[8.31E-6 – 1.03E-5]	1.44E-5	[9.82E-6 – 1.84E-5]
50%	1.64E-5	[1.36E-5 – 2.25E-5]	2.40E-5 – 3.30E-5*		2.99E-5	[2.38E-5 – 3.63E-5]
*Mixture 3c:14 components (ratio as defined in * *Table 4* *)*						
10%	5.00E-6	[3.67E-6 – 6.13E-6]	4.00E-6	[3.18E-6 – 4.63E-6]	6.68E-6	[3.47E-6 – 9.22E-6]
25%	9.79E-6	[8.64E-6 – 1.09E-5]	1.00E-5	[8.76E-6 – 1.09E-5]	1.54E-5	[1.07E-5 – 1.94E-5]
50%	1.96E-5	[1.67E-5 – 2.52E-5]	2.46E-5 – 3.51E-5*		3.15E-5	[2.52E-5 – 3.81E-5]
*Mixture 3d:14 components (ratio as defined in * *Table 4* *)*						
10%	3.07E-06	[2.63E-6 – 3.41E-6]	2.46E-6	[1.36E-6 – 2.93E-6]	3.17E-6	[1.14E-6 – 4.75E-6]
25%	7.15E-06	[6.01E-6 – 9.04E-6]	6.50E-6	[4.87E-6 – 7.24E-6]	8.22E-6	[4.35E-6 – 1.10E-5]
50%	1.85E-05	[1.40E-5 – 3.08E-5]	1.61E-5 – 2.65E-5*		1.84E-5	[1.28E-5 – 2.29E-5]
*Mixture 4∶13 components (ratio as defined in * *Table 4* *)*						
10%	2.12E-7	[1.56E-7 – 2.81E-7]	2.80E-7	[1.94E-7 – 3.60E-7]	3.30E-7	[1.70E-7 – 5.55E-7]
25%	6.52E-7	[5.33E-7 – 8.02E-7]	7.78E-7	[6.19E-7 – 9.02E-7]	8.42E-7	[5.46E-7 – 1.22E-6]
50%	1.77E-6	[1.47E-6 – 2.24E-6]	2.11E-6 – 2.51E-6*		1.98E-6	[1.52E-6 – 2.56E-6]

CA – Concentration Addition, IA – Independent Action, CI – Confidence Interval; All predictions statistically significant to the observed ECs are shown in bold; *Effect mixture concentration for effect levels higher than the lowest estimated compound maximal model asymptote are extrapolated either (i) by assuming no contribution of this compound to the overall mixture effect (toxic unit equals zero), or (ii) by setting the compounds’ toxic unit to a fixed level equalling the value at the mixture concentration producing an effect of 0.7*θ_max_ (see Table 3). The right side of the interval corresponds to (i) and the left side to (ii), defining the range of possible CA predictions.

#### Modulation studies (ESCREEN only)

Modulation studies comprised screening of individual modulators in low power experiments accompanied by parallel testing of an equimolar mixture of all modulators. This combination of low power screening and mixture testing provides the opportunity to assess a high number of chemicals (16 in this case) without a prohibitive amount of experimental effort. In this case the effects of 16 modulators could be evaluated using a total of 19 experimental plates (16 for screening and 3 for mixture testing).

To screen individual potential modulators, the concentration of a mixture of estrogenic chemicals (13 components, equieffective design) that evoked an approximately 50% response was selected as the baseline and each modulator was tested over a range of concentrations for their ability to increase (positive modulation) or decrease (negative modulation) the observed ESCREEN response. To normalize for small changes in the absolute value of the positive control and the control response to the selected REF_mix_ concentration, the response evoked by the REF_mix_ control on each plate was defined as the 50% effect and all modulator study results are normalized to that value.

A mixture of modulators (MODmix) was also tested and was composed with a fixed ratio of equimolar concentrations, since the activity of each modulator was not known at the time of designing the mixture.

### Concentration-response Analysis

Raw results from either in vitro assay were normalised by subtraction of the mean value of on-plate vehicle controls and then division by the mean value of on-plate positive controls. Experiments were performed on different days meaning that results for different dilution series and different batches of the test chemicals were obtained on different days. Each single chemical was tested in three or more independent experiments. Use of normalisation meant that data from different experiments could be reliably combined and compared, as discussed previously [12]. Data was expressed relative to the positive control, rather than as fold change from the vehicle response, for two reasons. Firstly, to anchor the response data to a meaningful response, namely the maximal response of the cognate ligand (estradiol), and secondly, to avoid spurious changes on the response axis due to the very low background estrogenicity which was achieved in vehicle controls in both assay systems. When the vehicle response is numerically small, chance variations that are not experimentally significant can nonetheless significantly affect the apparent fold change of positive test chemicals.

Data from multiple replicate experiments were pooled and statistical concentration response regression analyses were conducted for each single compound according to the best-fit approach [13]. Effect concentrations (EC_x_) were defined relative to the effects of the estradiol concentration used as the positive control, for example EC_50_ is the concentration producing an effect of 50% of the positive control (estradiol, 1 nM (ERLUX) or 25 nM (ESCREEN)).

### Prediction of Mixture Effects

Results from regression analysis for each test chemicals were used to predict the CA effect for fixed-ratio mixtures designed on 1) equi-effective concentrations (balanced design), 2) possible human serum levels (non-balanced design). The original mathematical formulation of CA that defines for a mixture composed of *n* components with concentration *c_1_* of the first component, *c_2_* of the second component, and *c_n_* for the n-th component a combination effect X is
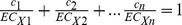
(1)Here, *EC_X1_*, *EC_X1_* …, *EC_Xn_* are the concentrations of the individual components that on their own produce the same effect *X* as the mixture [14], [15] The quotients *c_n_*/*EC_Xn_* are called toxic units and scale all compounds in the mixture relative to their toxicity. They can be interpreted as the contribution of the compound to the total mixture concentration that is expected to produce the mixture effect X. According to equation 1, a mixture effect for a pre-defined effect level X is described only implicitly. However, if the individual concentrations *c_i_* are expressed as relative fractions *p_i_* to the total mixture concentration *ECx_Mix_*, then equation 1 can be re-arranged to
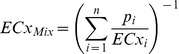
(2)which allows the direct calculation of the mixture concentration that is expected to produce the mixture effect X. To form a prediction curve, equation 2 is iterated through for various effect levels resulting in sets of effects and their associated predicted mixture concentration which are then plotted to produce a graph of the effect prediction.

Both equations 1 and 2 require knowledge of the concentration of each mixture component that on its own produces the effect magnitude under consideration. For this reason, these equations cannot be used for predicting mixture effects that exceed the maximal effect of the least efficacious component, because those effect concentrations cannot be defined. We have referred to these chemicals as ‘sub-maximal’ because they have an effect maxima that is lower than that of the positive control, estradiol. This situation is not uncommon for estrogenic compounds where dose-response curves with different maxima have been observed, for example in the ESCREEN [11]. To overcome this limitation, we developed a pragmatic solution that extrapolates the toxic units of sub-maximal mixture components to effect levels beyond their maximal efficacy. In short, the toxic unit of a sub-maximal component is fixed above a certain concentration in the mixture and used for all higher concentration in the mixture to calculate the mixture concentration for higher effect levels. Here we followed two approaches: by defining as cut-off value the concentration that produces an effect equaling 70% of the maximal model asymptote (θ_max_, see Tables 2 and 3), we used either (i) the associated toxic unit in the mixture prediction as fixed maximal contribution, or (ii) we set the toxic unit to zero (minimal contribution). These two worst-case calculations define a range of possible CA predictions, with the left side of the interval (higher effects) corresponding to (i) and the right side (lower effects) to (ii).

The composition of each tested mixture is listed in Table 4. The statistical uncertainty for mixture effects predictions was determined using the bootstrap method [16] and expressed as 95% confidence limits for the predicted mean estimate (Tables 5 and 6). Differences between predicted and observed effect doses were deemed statistically significant when the 95% confidence belts of the prediction did not overlap with those of the experimentally observed mixture effects.

The mathematical and statistical procedures used for calculating mixture effects according to independent action (IA) are described in [12]. Effects predicted by IA are included in the results tables for completeness (Tables 5 and 6), but only CA predictions are included in the figures for clarity and because the experimental situation used CA was deemed the more appropriate model.

## Results

### Single Chemical Testing

**Figure 1:**
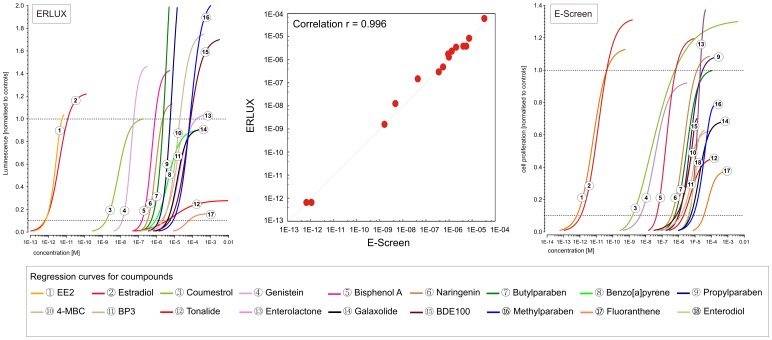
Concentration-response curves from single chemical testing and inter-assay correlation. Graphs show the results of testing single estrogenic chemicals in the ERLUX (left) or ESCREEN (right) assays. Each single chemical was tested in three or more independent experiments. Results are shown as the best fit regression model, for which details are provided Tables 2 and 3. Middle graph shows the correlation between EC10 values obtained in the ERLUX (y-axis) and ESCREEN (x-axis) assays.

Concentration response curves for the single components included in mixtures of estrogens are shown for the ERLUX (Figure 1, left; Table 2) and ESCREEN (Figure 1, right; Table 3) assays. Figure 1 (left graph) shows that chemicals tested in the ERLUX exhibited a wide range of potencies, for example EC_10_ values ranged from picomolar to high micromolar concentrations. The most potent chemicals tested were estradiol and ethinylestradiol, and the least potent was fluoranthene. Supramaximal responses (responses greater than the maximal response for the cognate ligand estradiol) were quite commonly observed in the ERLUX assay, for example for genistein (200% of estradiol maxima), bisphenol A (150%), butylparaben (300%) and benzophenone-3 (150%). Supramaximal effects were observed over the range of concentrations tested, i.e. they did not appear to be related to the potency of the chemical. The supramaximal effect varied between chemicals and could be up to around 300% of the maximal effect of estradiol, e.g. for butylparaben. Supramaximal effects are a known feature of the ERLUX assay [8], and are also observed in similar reporter-gene assays [17], [18].

Figure 1 (right graph) shows that chemicals tested in the ESCREEN exhibited a similarly wide range of potencies to the ERLUX. However supramaximal effects were not typically observed in the ESCREEN, instead a number of chemicals showed submaximal responses, e.g. they exerted a maximal effect that was substantially lower than that of estradiol, for example 4MBC and benzophenone-3 both showed clear maxima at effects levels of 60–70% that of estradiol.

#### Comparison of assays

Figure 1 (middle graph) shows a comparison of the 10% effect concentrations (EC_10_) determined for chemicals tested in the ERLUX and ESCREEN assays. Excellent correlation between the two assays was seen when compared at EC_10_ (r^2^ = 0.996).

**Figure 2:**
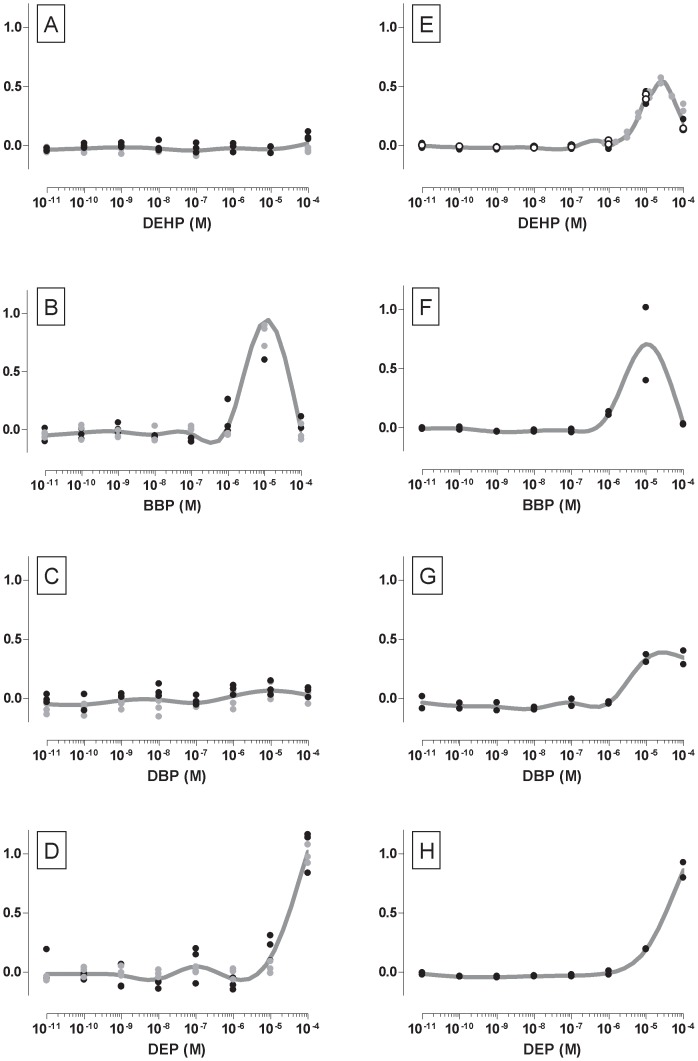
Different effects of phthalates in ERLUX and ESCREEN. Graphs show the results of testing four phthalates (DEHP, BBP, DBP, DEP) in the ERLUX (A–D) and ESCREEN (E–H) assays. Each phthalate was tested in two independent experiments in ERLUX (triplicate testing within assay) and three (DEHP) or one (BBP, DBP, DEP) experiments in ESCREEN (duplicate testing within assay).

Whilst most of the chemicals screened showed similar results in the two assays, differences were observed for phthalates and for benzo [a] pyrene. Four phthalates were tested in both assays: DEHP, BBP, DBP and DEP. In ERLUX we found that DEHP and DBP showed no estrogenicity whilst both BBP and DEP were estrogenic (activity seen at concentrations of 0.1–1 µM and above) (Figure 2). In the case of BBP there was evidence for toxicity within the dose-response analysis, and this appeared to occur close to the concentration at which estrogenicity was observed. In ESCREEN, we found that all four phthalates showed activity. At the highest concentrations shown in Figure 2, both DEHP and BBP showed a decline in response, which may indicate toxicity. The estrogenicity of DEHP occurred at concentrations greater than 1 µM, and toxicity was seen before a full estrogenic response was reached (toxicity began at 5 µM). Because of these differences between assays and, in some cases, the narrow margin between effect and apparent toxicity, phthalates were not included in the mixtures of estrogens, however they were examined as potential modulatory components, see “Modulator studies” section below.

Benzo [a] pyrene (BaP) was tested alone in ERLUX prior to being examined as an effect modulator, and was found to be active (see Figure 1). This was unexpected, based on the literature, for example [19], and meant that BaP was included in the mixtures of estrogenic chemicals for testing in the ERLUX (see “Mixture Studies” section next). Conversely, BaP was not active when tested alone in the ESCREEN and therefore it was not included in any of the mixtures of active estrogens tested in the ESCREEN, however BaP was tested as an effect modifier in the ESCREEN (see “Modulator studies” section below).

### Mixture Studies (“Balanced” Design: Fixed Ratio Effective Concentration)

**Figure 3:**
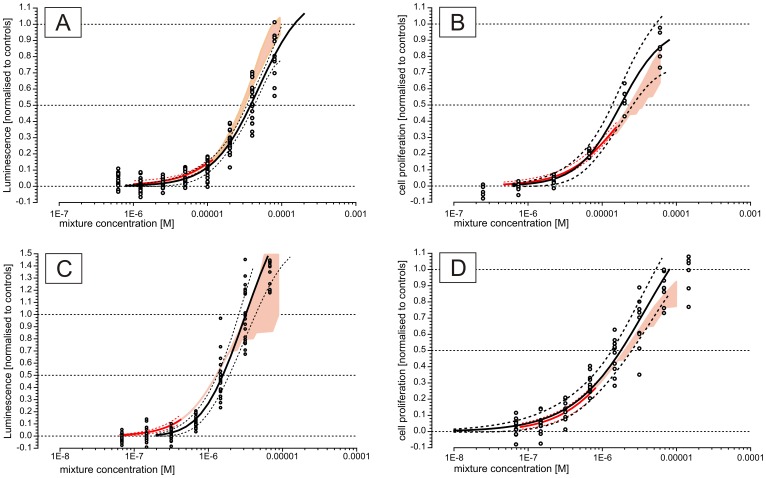
Predicted and observed effects of mixtures. A) 17 component mixture at fixed mixture ratio proportional to the individual EC10’s (ERLUX, Mixture1 (Table 4)). B) 16 component mixture at fixed mixture ratio proportional to the individual EC25’s (ESCREEN, Mixture3a (Table 4)). C) 14 component mixture at fixed ratio proportional to approximate human tissue concentrations (ERLUX, Mixture 2 (Table 4)). D) 13 component mixture at fixed ratio proportional to approximate human tissue concentrations (ESCREEN, Mixture 4 (Table 4)). Each graph shows experimental data (dots) with best fit regression curves (solid black lines) and their 95% confidence belts (dotted black lines). Prediction curves according to concentration addition are shown as red solid line, with approximate 95% confidence intervals as dotted red lines. The use of extrapolation to extend the range of CA is shown by a pale red band, which is delimited by worse-case upper and lower assumptions (see text for more details).

In the ERLUX assay, we tested an equieffective mixture of 17 active components (Mixture 1 in Table 4; fixed ratio of EC_10_ levels) over an effect range from 0 to 100% effect. Testing revealed good agreement with CA, as shown by the experimental data overlapping the predicted CA line (Figure 3A). Figure 3A also shows the use of extrapolation to extend the predictive rage of the CA model (See “Methods” section). Without extrapolation, the CA model equations limit the predicted effect to the lowest maximal effect of any of the tested components, which can even be as low as 10–20% for some chemicals, see Figure 1 and Tables 2 and 3.

In the ESCREEN a 16 component mixture (Mixture 3a in Table 4; fixed ratio of EC_25_ levels), differing from the mixture tested in ERLUX only by the omission of BaP and the use of EC_25_s rather than EC_10_s to set the fixed mixture ratio, showed good agreement with the CA prediction (Figure 3B). As was done in the ERLUX, extrapolation was also used to extend the CA prediction for the ESCREEN data.

Three additional balanced mixtures were also tested in the ESCREEN and were composed as listed in Table 4 (mixtures 3b, 3c, 3d). Mixtures 3b and 3c omit ethinylestradiol (3b) and estradiol (3c) and were included to examine a potential small deviation from additivity hypothesized to be due to the steroid nature of these two chemicals which was previously observed [5]. In the event, no such deviation was observed in our studies and these mixtures simply constitute testing of differently composed mixtures whose effects showed good conformance to CA (Table 6). Mixture 3d was included in order to test a mixture containing the brominated flame retardant (BDE100), which is a potentially important human estrogen [20]–[22]. Mixture 3d also showed good conformance to CA (Table 6).

### Mixture Studies (“Non-balanced” Design: Fixed Ratio in Proportion to Human Exposure Levels)

In order to examine the robustness of the use of CA to predict mixture effects we repeated mixture studies in both assays using a second mixture design, which we have termed a ‘non- balanced’ design to contrast it with the balanced, equieffective design used so far (Figure 3A, 3B). For example, in a 17 component, equieffective mixture each component is expected to contribute one seventeenth of the mixture effect. The balanced design gives the best chance of seeing a deviation from a mixture effect since a change in the contribution of any one single component is equally likely to be detected, however this situation is not likely in realistic mixture scenarios. The ‘non-balanced’ design uses a fixed ratio of concentrations that was initially based on the approximate tissue concentrations that have been reported to be found in human tissues, mostly serum, and was then refined to prevent any single component, such as estradiol, from dominating the mixture effect. It is not our intention that this should be considered as a comprehensive model for the human exposure scenario, since the database for tissue concentrations is incomplete, dominated by certain frequently measured compounds and is a massive simplification of the complexity of human exposure (ignoring at least temporal and geographical variations in exposure).

The mixtures used similar components to the mixtures designed with a fixed ratio of equieffective concentrations, but with some omissions: ethinylestradiol was removed because it has different exposure consideration to almost all of the other components due to its use as a pharmaceutical; galaxolide and tonalide were not included for technical reasons (a temporary lack of availability of the pure compounds).

**Figure 4:**
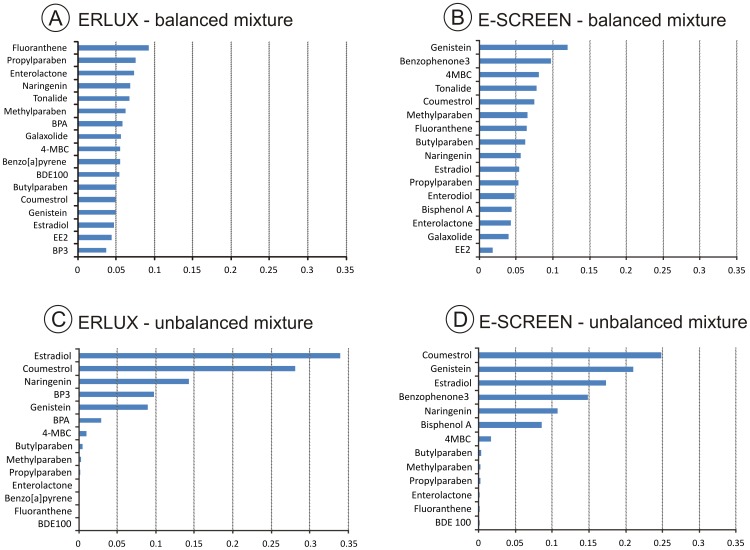
Distribution of toxic units. Each graph shows the distribution of toxic units as predicted by CA at the EC10 level for mixtures of estrogenic compounds tested in the ERLUX and ESCREEN. A) 17 component mixture at fixed mixture ratio proportional to the individual EC10’s (ERLUX, Mixture1 (Table 4)). B) 16 component mixture at fixed mixture ratio proportional to the individual EC25’s (ESCREEN, Mixture3a (Table 4)). C) 14 component mixture at fixed ratio proportional to approximate human tissue concentrations (ERLUX, Mixture 2 (Table 4)). D) 13 component mixture at fixed ratio proportional to approximate human tissue concentrations (ESCREEN, Mixture 4 (Table 4)).

Figures 3C and 3D show the results of testing mixtures with a ‘non- balanced’ design in the ERLUX (fourteen components, Figure 3C, Mixture 2 (Table 4)) and in the ESCREEN (thirteen components, Figure 3D, Mixture 4 (Table 4)). The distribution of toxic units indicates how balanced a mixture design is, and these distributions are shown in Figure 4 for the same four mixtures shown in Figure 3. Figure 4C and D show clearly that the non-balanced design results in a greater contribution from 6 of the components, and that the contributions are heavily skewed. In contrast, for mixtures with a balanced design the bars in the TU distributions are of a similar length (Figure 4A, B). In both assays, the effects of these less-balanced mixtures was well predicted by CA. Toxic unit distributions (Figure 4C, 4D) showed that the components contributing most to these mixture effects were estradiol, coumestrol, naringenin, bisphenol A, genistein and benzophenone-3. It is important to note that the relative contribution assigned to this list of components is due to both the levels reported to occur in human tissue and to the mixture design. Consequently this approach could be used to prioritise components for regulatory attention and/or action only if reliable human exposure levels were used as the input.

### Modulation Studies

Modulation studies were carried out only using the ESCREEN assay because we considered that a cell proliferation assay would have more potential to show any modulation than a reporter-gene assay in which the link between chemicals and their effects is engineered and presumed to be more direct.

In these modulator studies, we examined 16 single chemicals (listed in Table 7) and an equimolar mixture of all 16 components for their ability to modulate the effects of a reference mixture (REFmix) which contained 14 food additives and contaminants (Table 1, Mixture 3d). The concentration of REFmix was chosen to evoke a response of around 50–60% in the ESCREEN, which gave the opportunity to detect any increase in effect (positive modulation) as well as any decrease (negative modulation). Single chemicals were tested individually in single experiments (screening) and then a mixture composed using a fixed ratio of equal molarities was subjected to repeated testing.

**Table 7:** Observations of estrogenicity or toxicity for chemicals screened as potential modulators.

Name of potential modulator	Observed modulation^a^	Signs of estrogenicity^b^	Signs of toxicity^c^
2-amino-1-methyl-6-phenylimidazo [4,5-b] pyridine (PhIP)	Possible negative	None	None
2-Amino-3,8-dimethylimidazo [4,5-f] quinoxaline (MeIQx)	None	None	None
Benzo [a] pyrene (BaP)	Clear negative	None	Possible toxicity at 3 µM and greater
Butyl benzyl phthalate (BBP)	None	Active at 1 µM or higher	Toxic at 100 µM
Butylated hydroxyl anisole (BHA)	Possible negative	None	None
Butylated hydroxytoluene (BHT)	Possible negative	None	None
Cadmium chloride (CdCl_2_)	Clear negative	None	
Di butyl phthalate (DBP)	Possible negative	Active at 10 µM or higher	None
Di ethyl hexyl phthalate (DEHP)	None	Active at 1 µM or higher	Toxic at 50 µM and greater
Di ethyl phthalate (DEP)	None	Active at 10 µM or higher	None
Lead nitrate (Pb(NO_3_)_2_)	Possible negative	None	None
Mercury chloride (HgCl_2_)	None	None	Possible toxicity at 10 µM and greater
PCB #126	Clear negative	Active at 10 µM or higher	None
PCB #153	None	None	None
PCB #180	None	None	None
PCB #8	None	None	None

a results from modulator screening studies were classified as ‘*clear negative*’ (reduction in effect of REFmix at multiple concentrations showing an approximately sigmoid dose-response), ‘*possible negative*’ (reduction in effect of REFmix at a single concentration or multiple concentrations with a apparent linear concentration-response relationship) and ‘*none*’ (no indication of a negative or positive effect).

b ‘*Active’* indicates a positive signal in ESCREEN (estrogenicity), clearly distinguishable from assay variability and noise and usually supported by a dose-response (i.e. not reliant on data from only a single concentration).

c ‘*Possible toxicity’* indicates a decrease in value for treated wells below that of vehicle controls, this signal is small so the assignment of toxicity is not certain; ‘*Toxic’* indicates a reduction in signal evoked by increasing concentrations above those at which a chemical showed activity (estrogenicity).

Potential modulators were selected from a pool of previously tested chemicals that were of interest due to their presence in food and that had the potential for endocrine disruption based on the literature. Potential modulators included both those found to have estrogenicity at the higher end of the tested range (>1 µM, usually 10–100 µM; phthalates and PCB126) and those that were not estrogenic when tested alone (heavy metals, antioxidants, heterocyclic amines, PCBs, poly aromatic hydrocarbons).

#### Effect of a mixture of 16 potential modulators

The 16 potential modulators were combined to make a ‘mixture of modulators’ (MODmix). The MODmix was designed using a fixed ratio of equimolar concentrations, rather than a fixed ratio of equi-effective levels because it was not known beforehand whether the modulators would have a common effect (for example some may have increased the REFmix effect whilst others may have reduced it) and because the commonalities between the modulators are expected to be fewer than, for example, a mixture of estrogenic compounds which all have estrogenicity in common and which effect can thus be used as the basis for equieffective designs.

**Figure 5:**
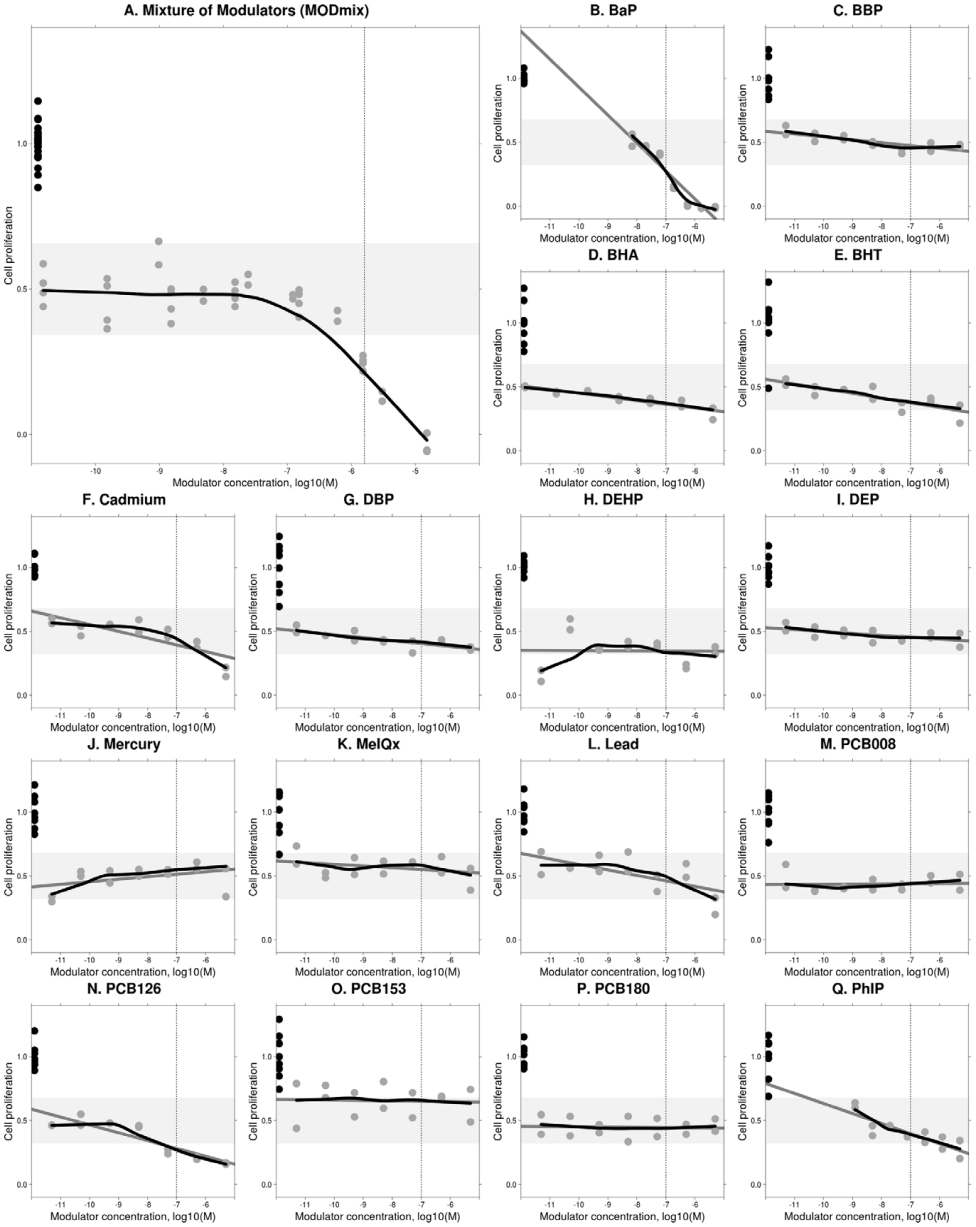
Results of modulation studies. Each graph shows the effect of a mixture of 16 modulators (A) or one of 16 individual modulators (B–Q), concentration indicated on the x-axis, on the ESCREEN response evoked by a reference mixture of 14 estrogens (Mixture 3d, Table 4). Experimental results are shown as grey circles representing each replicate (duplicate testing within the assay) and obtained in three independent (A) or one (B–Q) experiment (s). Experimental results were normalized by setting the value observed for the REF_mix_ alone, the concentration of which was selected to evoke a concentration of ca. 50%, to 0.5. The positive control values (black circles, eight replicates per experimental plate, 25 nM estradiol) are indicated adjacent to the y-axis. A horizontal grey strap indicates the approximate range of values observed using the positive control values on all experimental plates but centered at 0.5 to give a visual indication of the random noise present in the data. A black line indicates a LOESS spline fit to the data (A–Q) and a grey line indicates linear regression fit to the data for individual modulators (B–Q). A vertical dotted line is drawn at a mixture concentration of 1.6 µM (A) or at the concentration of each modulator present at that mixture concentration (0.1 uM, B–Q). Visual inspection of the individual modulator results (B–Q) was used to identify possible modulation, i.e. when the experimental data appears to deviate from the linear regression, or when the linear regression is not horizontal, and when the magnitude of the modulation is outside the variation expected in the assay.

Modulation studies using MODmix showed a clear negative modulation at a mixture concentration of 1.6 µM (i.e. which contains 16 modulators each present at 0.1 µM) and 16 µM, but not at 0.16 µM or lower (Figure 5A).

#### Effects of single modulators

Screening level data from studies in which 16 potential modulators were studied individually is presented in Figure 5B–Q. Data on estrogenicity and toxicity for these chemicals is listed in Table 7.

Considering the 16 potential modulators studied individually (Figure 5B–Q): a *clear* negative modulation was shown by three chemicals, which were benzo [a] pyrene, cadmium chloride and PCB126. A *possible* negative modulation was shown by five chemicals (lead nitrate, PhIP, DBP, BHA and BHT) whilst no indication of negative modulation was seen for eight chemicals (DEHP, mercury chloride, PCB008, PCB153, PCB180, MeIQx, BBP and DEP). None of the potential modulators showed any indication of a positive modulation.

We note that the distinction between clear and possible modulation is somewhat arbitrary due to the low power of these screening experiments and, for the same reason, strong conclusions of no effect cannot be drawn. Three compounds were considered to show a clear negative effect: PCB126 began to suppress the ESCREEN response to the REF_mix_ at concentrations between 1–10 nM, 50% suppression was reached at 100 nM, and the suppressive effect appeared to plateau at that level up to concentrations of 10 µM. When tested alone, PCB126 was estrogenic in ESCREEN at 10 µM. Cadmium chloride (CdCl2) began to suppress the REF_mix_ effect at around 10 nM, reached 80% suppression at 10 µM, and the trend in the data suggests that complete suppression would have been reached if higher concentrations were tested. Benzo [a] pyrene began to suppress the effect of REF_mix_ at concentrations between 10 and 100 nM, and complete suppression was reached by 1 µM.

A quantitative assessment of whether the overall modulation conformed to CA, based on the modulation observed for the individual component chemicals, was not made due to the screening nature of the individual chemical data. However qualitative comparisons of the concentrations at which negative modulation was observed (for example, comparing the vertical dotted line in Figure 5A versus Figure 5B–Q) suggests that the effects were consistent with additivity as the mixture showed negative modulation only when the concentration of the components was in the range at which certain of the individual components were themselves active (for example BaP, Figure 5B). Consequently, dramatic synergies or antagonisms can be qualitatively ruled out.

## Discussion

The mixture studies reported in this paper show that the CA model provided good predictions of multicomponent mixture effects in both the ERLUX and ESCREEN assays. We believe our mixture study in ERLUX is the largest mixture studied in this system published to date, and the first to test a non-balanced design. We compared two types of mixture design, described as balanced and non-balanced and illustrated by the resulting toxic unit distributions (Figure 4). Our results showed that, for either design, CA was a suitable predictor of effects, and thus CA may be suitable for use in modeling the likely effects of mixtures and reducing the need to experimentally test every possible mixture. These observations support previous findings for mixtures. The question of how relevant the exact non-balanced design is to the human exposure scenario will require better data on both human tissue levels and the relationship between in vitro estrogenicity and gross effects.

We chose to study mixtures in parallel in both the ESCREEN and ERLUX assays, however in neither assay was there any evidence of a deviation from the effect predicted by CA. The lack of a difference between the ERLUX and ESCREEN assays may be because the assays are not as different as we originally hypothesised, or because no deviation was observed in either case. It might have been expected that, if a deviation had occurred, it would be observed in the more complex ESCREEN and not in the engineered ERLUX assay. The dose-response curves obtained in the two assays were very similar and showed almost perfect correlation (Figure 1).

Studies of potential modulation of the effect (ESCREEN) of a mixture of estrogenic chemicals revealed negative modulation by a mixture of 16 modulators, with clearest indications for a role in this effect for 3 of the modulators (PCB126, CdCl2, BaP). This fraction (approximately 1 in 5, or 20%) of potential modulators applies only to this nonrandom sample of chemicals and should not be directly extrapolated to the wider chemical ‘world’. No indications for positive modulation were seen for any chemical. A preliminary analysis suggested that the observed negative modulation was also predictable by the CA model, although the low power and screening nature of these studies should be considered before drawing strong conclusions.

Frische *et al.* have previously examined the modulation of the estrogenicity of either estradiol or a ternary mixture of estradiol, estrone and estriol using a genetically engineered yeast estrogen screen [23]. They observed negative modulation by 2,4-dinitroaniline (organic solvent) and cycloheximide (antibiotic) and no effect of mercury chloride of DMSO (organic solvent). Interestingly, a positive modulation, or synergy, was observed for LAS-12 (a surfactant) but only at concentrations deemed ‘slightly toxic’ [23]. Our results, for sixteen different potential modulators, are thus consistent with the general picture observed by Frische *et al.,* in that typically either negative modulation or no effect was the observed outcome. We have built on the results of Frische *et al.* by using a non-engineered, mammalian assay system in which interactions might be more readily extrapolated to the in vivo mammalian situation, by using a much larger reference mixture (14 rather than 1 or 3 components) and by testing the ‘double’ mixture situation in which a mixture of modulators was found to negatively modulate the effects of a mixture of estrogens.

One possible explanation for the apparent negative modulation seen in our studies is the occurrence of frank toxicity or so-called toxic masking [23]. Testing for toxicity in parallel with the ESCREEN assay is not straightforward and was not routinely done. Complications include the low initial cell seeding density and that substantial cell proliferation occurs within the assay if estrogens are present but does not occur when estrogenic signals are absent. A standard cell viability assay, the MTT assay, could not be performed with the initial cell density (signal is too low) and assay duration of the ESCREEN (possibility of significant cell growth confounds assessment of cell viability). Performing an MTT assay with strikingly different parameters to those used in the ESCREEN was considered likely to remove the relevance of the cytotoxicity data to the estrogenicity data, and was therefore not done.

However, although direct cytotoxicity testing was not appropriate, a number of observations suggest that the negative modulation reported here should not be discounted as toxic masking. 1) Negative modulation was not seen for all chemicals of a similar type, for example cadmium chloride showed negative modulation whilst mercury chloride and lead nitrate did not. Interestingly, the estrogenicity or anti-estrogenicity of cadmium chloride has been the subject of much attention in the scientific literature and appears to have a complex nature that cannot be simply assigned to direct activity at an estrogen receptor [24]–[26]. 2) The concentrations at which negative modulation was seen were not extremely high and the effects began in the high nanomolar concentration range. 3) PCB126 showed negative modulation beginning at concentrations around 10 nM, however at higher concentrations in the range of 10 µM actual estrogenicity was shown (when PCB126 was tested alone) indicating that toxicity is not likely at the intermediate concentrations where negative modulation was observed. PCB126 also showed a clear plateau in its inhibitory effect (negative modulation) whilst frank toxicity would be expected to result in full inhibition and to not show a plateau in effect. 4) CdCl2 began to negatively modulate the estrogenic response at around 10 nM and the effect spanned several orders of magnitude, whereas frank toxicity might be expected to show a steeper dose-response relationship. 5) BaP showed full inhibition at concentrations around 1 µM, however the residual baseline in the assay was not abolished (which would be seen as a sub-zero response below zero, Figure 5B), as can occur when there is frank toxicity. This lack of effect on the baseline provides an indication for a lack of toxicity, albeit somewhat crude. Interestingly, BaP was estrogenic when tested alone in the ERLUX assay (but not when tested alone on in the ESCREEN assay) possibly indicating that, like cadmium, BaP is capable of an interaction with the estrogen system that is yet to be fully understood.

There are some indications in the literature of potential mechanisms for modulation for all three of the compounds that showed clear negative modulation, including observed effects on estrogen signaling that could be subtle or indirect (CdCl_2_ [24], BaP [27]) and effects on metabolism such as AhR agonism or cytochrome P450 enzyme induction (PCB126, BaP [27]; BaP [28]).

The modulation testing process we adopted provides a scalable compromise approach to testing compared to an exhaustive combinatorial approach that would only be feasible in a high-throughput, automated system, and for which the benefits may not justify the high costs in time and experimental resources. The use of parallel low power screening and mixture testing increases the strength of the observations because they can be used to cross-validate each other. The definition of modulation, or of modulator chemicals, is not trivial, since requiring that each potential modulator has a complete absence of the activity being examined for modulation requires testing at high concentrations at which assay function can be impaired and technical issues may confound interpretation. A clear positive modulation would be less equivocal than a clear negative modulation, for which toxic masking is an alternative explanation and must be explicitly considered. Ideally chemicals would be profiled both for their activity on multiple receptors or systems, and for modulation of those effects. The methods used here may be suitable to allow wider consideration of modulation that has been commonplace to date.

We have shown that negative modulation can be observed in the ESCREEN and provide indications that the extent of this modulation should be quantitatively predicted by using information from testing of the single components. The possibility of modulation should be considered when attempting to predict the effects of mixtures in real human exposure scenarios which are unlikely to be limited to only chemicals with one defined effect. The observed negative modulation can be considered to be a large, convincing deviation from CA/additivity. Our results show that *in vitro* models such as the ESCREEN could be useful to explore significant deviations that may be encountered when the type of chemicals included in mixture studies is expanded, and that further studies may be relevant as a precursor to the design of similar *in vivo* studies. Finally, the results of our mixture studies support the growing consensus that conformance to additivity (CA) should be expected for multicomponent mixture of estrogens, but consideration of potential modulations is also necessary to accurately predict the likely outcomes when complex, mixed exposure scenarios are examined.
